# Nanographene oxide-methylene blue as phototherapies platform for breast tumor ablation and metastasis prevention in a syngeneic orthotopic murine model

**DOI:** 10.1186/s12951-018-0333-6

**Published:** 2018-01-30

**Authors:** Mayara Simonelly Costa dos Santos, Ana Luisa Gouvêa, Ludmilla David de Moura, Leonardo Giordano Paterno, Paulo Eduardo Narcizo de Souza, Ana Paula Bastos, Emanuel Adelino Medeiros Damasceno, Fabiane Hiratsuka Veiga-Souza, Ricardo Bentes de Azevedo, Sônia Nair Báo

**Affiliations:** 1 Electron Microscopy Laboratory, Institute of Biological Sciences, University of Brasilia, Brasília, Brazil; 2 Nanobiotechnology Laboratory, Institute of Biological Sciences, University of Brasilia, Brasília, Brazil; 3 Laboratory of Research on Polymers and Nanomaterials, University of Brasilia, Brasília, Brazil; 4 Laboratory of Software and Instrumentation in Applied Physics and Laboratory of Electron Paramagnetic Resonance, Institute of Physics, University of Brasilia, Brasília, Brazil; 5 Embrapa Pigs and Poultry, Brazilian Agricultural Research Corporation, Concórdia, Santa Catarina Brazil; 6 Nucleus of Pathological Anatomy, Regional Hospital of Taguatinga, Taguatinga, Brazil; 7 School of Ceilandia, University of Brasilia, Brasília, Brazil; 8 Laboratory of Protein Chemistry and Biochemistry, Institute of Biological Sciences, University of Brasilia, Brasília, Brazil

**Keywords:** Photodynamic therapy, Photothermal therapy, Graphene oxide, Photosensitizer dye, Bioluminescence, Tumor regression and metastasis avoidance

## Abstract

**Background:**

In the photodynamic therapy (PDT), the photosensitizer absorbs light and transfers the energy of the excited state to the oxygen in the cell environment producing reactive oxygen species (ROS), that in its turn, may cause cell damage. In the photothermal therapy (PTT), light also is responsible for activating the photothermal agent, which converts the absorbed energy in heat. Graphene oxide is a carbon-based material that presents photothermal activity. Its physical properties allow the association with the photosensitizer methylene blue and consequently the production of ROS when submitted to light irradiation. Therefore, the association between nanographene oxide and methylene blue could represent a strategy to enhance therapeutic actions. In this work, we report the nanographene oxide-methylene blue platform (NanoGO-MB) used to promote tumor ablation in combination with photodynamic and photothermal therapies against a syngeneic orthotopic murine breast cancer model.

**Results:**

In vitro, NanoGO-MB presented 50% of the reactive oxygen species production compared to the free MB after LED light irradiation, and a temperature increase of ~ 40 °C followed by laser irradiation. On cells, the ROS production by the nanoplatform displayed higher values in tumor than normal cells. In vivo assays demonstrated a synergistic effect obtained by the combined PDT/PTT therapies using NanoGO-MB, which promoted complete tumor ablation in 5/5 animals. Up to 30 days after the last treatment, there was no tumor regrowth compared with only PDT or PTT groups, which displayed tumoral bioluminescence 63-fold higher than the combined treatment group. Histological studies confirmed that the combined therapies were able to prevent tumor regrowth and liver, lung and spleen metastasis. In addition, low systemic toxicity was observed in pathologic examinations of liver, spleen, lungs, and kidneys.

**Conclusions:**

The treatment with combined PDT/PTT therapies using NanoGO-MB induced more toxicity on breast carcinoma cells than on normal cells. In vivo, the combined therapies promoted complete tumor ablation and metastasis prevention while only PDT or PTT were unable to stop tumor development. The results show the potential of NanoGO-MB in combination with the phototherapies in the treatment of the breast cancer and metastasis prevention.

**Electronic supplementary material:**

The online version of this article (10.1186/s12951-018-0333-6) contains supplementary material, which is available to authorized users.

## Background

Breast cancer is the second most common cause of death in women worldwide. Its high incidence, aggressiveness, and low prognosis arouse interest in the health field [1, 2]. The currently available treatments, including surgery, radiotherapy, immunotherapy, hormonal treatment and mainly chemotherapy, may not promote complete tumor ablation, cause damage to normal cells, leading to adverse side-effects [3].

Phototherapies, such as photodynamic (PDT) and photothermal (PTT) therapies, have emerged as promising alternatives owing to their specificity, low systemic toxicity, and low invasiveness in comparison with other therapies [4–8]. In addition, it has been shown that these therapies can be used in combination with other available therapies, as well as with targeted drug delivery systems, which can be more efficient in overcoming tumors [9, 10]. To be effective, PDT needs three components, oxygen (O_2_), a photosensitizer (PS) and light of specific wavelength. PS absorbs the energy of photons with a specific wavelength to create an excited state that decays to the ground state, transferring energy to O_2_ molecules present in the cell environment. This changes on their electronic state to convert them into reactive oxygen species (ROS), such as singlet oxygen (^1^O_2_), triplet oxygen (^3^O_2_) or superoxide anion (O^2^˙^−^). These ROS, mainly ^1^O_2_, are responsible for causing damage and death in target cells due to the oxidative stress [5, 11]. PTT usually uses red and near-infrared (NIR) visible light, which is near the tissue transparency window, to excite the photothermal agent [7]. In PTT, the light absorbed is converted into heat through a non-radioactive decay. The resulting hyperthermia in cells or tissues leads to tissue coagulation, irreversible cell damage and necrosis [11]. A further advantage is the restricted damage to a specific site and, therefore, the preservation of healthy tissue [12]. However, the greatest limitation of this approach is the light penetration depth in the tissues. As a result, PTT is preferably performed in smaller tumors and in which ones that remain superficial in the body. Due to this limitation, a non-depth tumor like the breast tumor is suitable to assess the efficiency of this therapy in heating the target tissue and subsequently in promote cell death [7].

Graphene is the smallest subunit of graphite. It features a one-atom thick sheet-like structure of sp^2^ carbons that gives it outstanding mechanical, thermal, and optoelectronic properties [12–14]. However, due to its high hydrophobicity, it is difficult to be implemented as a diagnosis-therapy platform in biological environments. These shortcomings can be overcome by its oxidized form, known as graphene oxide (GO), which is hydrophilic and can be produced at large scale by an inexpensive oxidative exfoliation of graphite. GO is endowed with different oxygen-based functionalities, including ether, epoxy, alcohol and carboxylic acid, which may serve as anchoring sites for loading a myriad of biomedical agents, such as antibodies, PS, deoxyribonucleic acid, and radionuclides (e.g. copper-64) [13, 15, 16]. Furthermore, NanoGO sheets, displaying lateral dimensions below than 100 nm, absorbs strongly in the near-infrared (NIR) visible light range and has been used as a photothermal agent for cancer treatment. This additional property is also allied to its high surface area (~ 2630 m^2^ g^−1^), which is suitable for loading of both hydrophilic and hydrophobic molecules as photosensitizers—which together may exhibit combined photodynamic and photothermal properties in a single biomedical nanodevice. Furthermore, the low in vitro and in vivo toxicity makes NanoGO an excellent NIR-based PTT agent [7, 11, 15, 17].

The hydrophilic molecule methylene blue (MB) is an inexpensive dye that exhibits a wide light absorption window (600–900 nm), with a peak at 660 nm, and singlet oxygen species quantum yields of 0.52. It also presents low dark toxicity, it is FDA-approved to be used in humans in the treatment of methemoglobinemia and has been used as a promisor photosensitizer for PDT in the treatment of viruses, bacteria and cancer cells [11, 18–21]. All these properties make it a good photosensitizer for use in photodynamic therapies. Nevertheless, MB chemical modification in its inactive form, leukomethylene blue, hinders reactive oxygen species production when administered in biological systems. Thus, this barrier could be overcome by MB loading in a nanoplatform as GO, what could grants to MB a variety of new desirable properties such as protection against biological environment degradation, enhanced delivery, longer circulation time and bioavailability [11, 12, 22].

Previous studies have shown that NanoGO conjugated with different PS are effective PDT/PTT agents against different tumors [23, 24]. Here the tumor model comprises a murine mammary carcinoma of bioluminescent cells 4T1-Luc, which mimic tumor growth and metastasis of stage IV human breast cancer and, therefore, may be more suitable for full exploration of the nanoplatform NanoGO-MB as a PDT/PTT agent [11, 14, 25–27]. The present study aims to address this issue by evaluating the efficiency of a combined PDT/PTT agent based on NanoGO-MB in vitro and in vivo in a syngeneic orthotopic tumor model induced in female BALB/c mice.

## Results and discussion

### Preparation and characterization of the NanoGO-MB platform

NanoGO was functionalized with carboxylic acid groups and loaded with MB. Table 1 presents hydrodynamic diameter (HD), polydispersity index (PDI), and zeta potential (ZP) of all GO-based samples.

**Table 1:** Nanomaterials properties

Samples\diluent	HD (nm)	PDI	Zeta potential (mV)
GO\water	254.4 ± 5.0	0.250 ± 0.060	− 43.0 ± 1.11
NanoGO\water	103.0 ± 0.5	0.242 ± 0.240	− 41.7 ± 2.75
NanoGO-Pluronic F127\water	122.2 ± 3.9	0.370 ± 0.320	− 36.5 ± 0.90
NanoGO-MB (0.22 µm-filtered)\water	112.5 ± 8.45	0.319 ± 0.340	− 46.2 ± 1.12

*GO* graphene oxide, *NanoGO* nanographene oxide, *MB* methylene blue, *HD* hydrodynamic diameter, *PDI* polydispersity index

In brief, the starting material, GO (Table 1), exhibits HD of 254.4 ± 5.0 nm, PDI of 0.250 ± 0.060, and zeta potential of − 43.0 ± 1.1 mV. After the carboxylation process and ultrasonic stirring, the HD was reduced reaching the size of 103.0 ± 0.5 nm (Table 1). In addition, the as-produced NanoGO displays a negative zeta potential. The addition of Pluronic F127 leads to a slight increase of HD and of the zeta potential, which turned less negative. Finally, the NanoGO-MB sample exhibited HD of 112.5 ± 8.45 nm and zeta potential of − 46.2 ± 1.12 mV (Table 1). As described elsewhere, Pluronic F127 acts partially masking the negative surface charges of the NanoGO [11]. Pluronic F127 interacts with the NanoGO via hydrophobic interactions, which takes place between hydrophobic propylene oxide (PPO) blocks and remaining graphitic regions in the NanoGO sheets. The use of Pluronic F127 is to ensure the colloidal stability of the NanoGO-MB platform and also to provide sufficient biocompatibility with the physiological medium. Despite the hydrophobic nature of PPO blocks in Pluronic, MB is not expected to interact with it as stronger as it does with NanoGO. Actually, at the physiological condition tested (PBS, pH 7.4) methylene blue (MB) interacts with graphene oxide mainly through electrostatic interactions, since MB is in its cationic form while NanoGO is anionic due to carboxylate groups, as confirmed by its zeta potential (− 46.2 ± 1.12 mV). Nonetheless, hydrophobic pi-stacking cannot be ruled out because other authors have previously identified such an interaction in the Nano-GO/MB system [28]. The colloidal stability of different platforms, NanoGO, NanoGO + Pluronic, NanoGO-MB, and NanoGO-MB + Pluronic, prepared in different media (deionized water, phosphate buffer saline, pH 7.4, and Dulbecco’s Modified Eagle’s Medium (DMEM) with 10% of FBS was ascertained by measuring their hydrodynamic diameter with dynamic light scattering. The measurements were taken after right after of samples preparation. The respective data are provided in Additional file 1: Table S1. The samples prepared in DMEM showed smaller hydrodynamic diameter and, therefore, higher stability. The enlarged size of samples prepared in PBS reflects the charge screening in NanoGO by the high concentration of salts in this buffer. However, it is important to point out that the NanoGO + Pluronic + MB was used right after its preparation so that long term monitoring was not necessary.

**Fig. 1:**
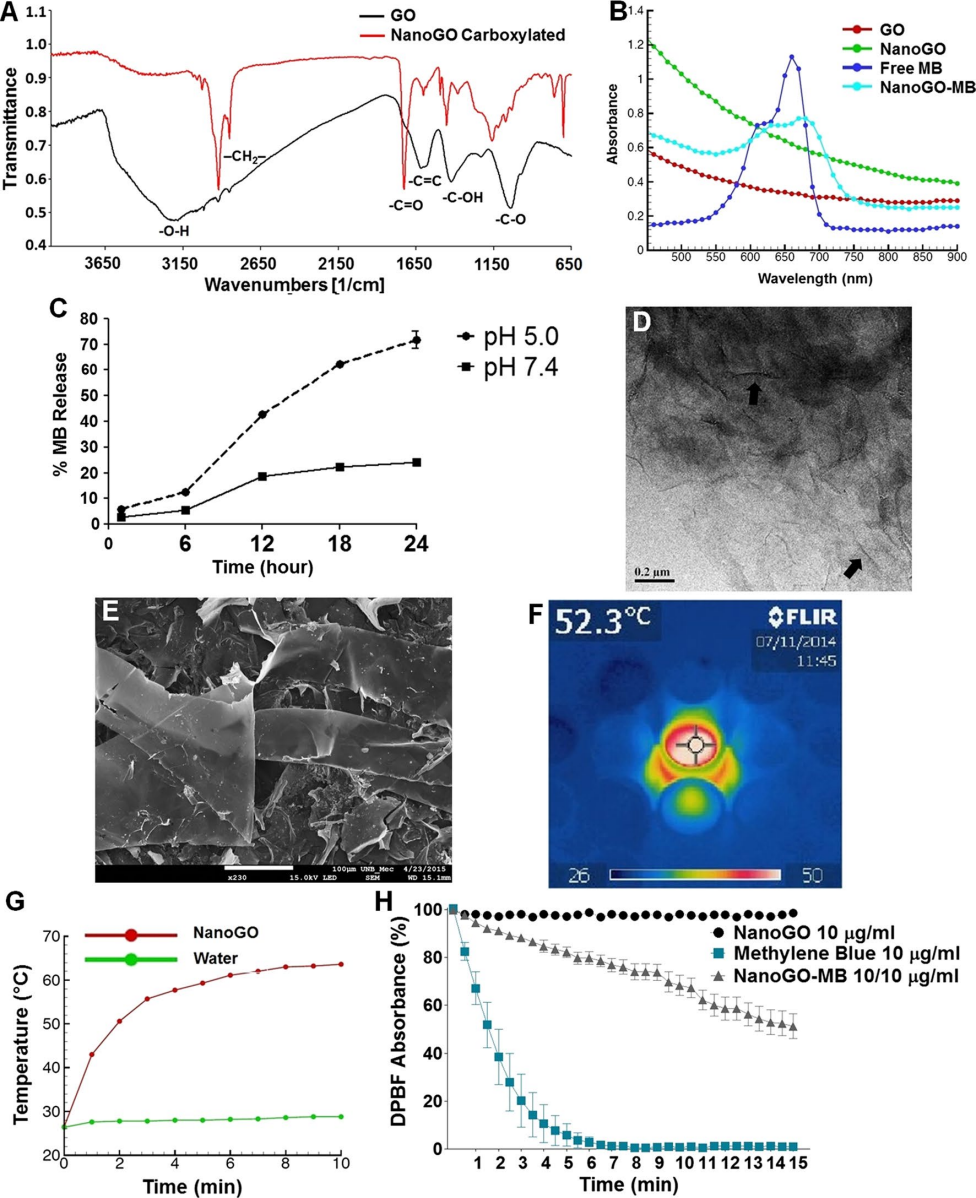
NanoGO and NanoGO-MB ultrastructure and characterization. **A** Oxygenated groups of GO and carboxylated NanoGO indicated on the FTIR with the prominence of the carboxylic acid group in 1728 cm^−1^ in carboxylated GO (red), suggesting successful carboxylation. **B** UV–Vis of samples showing the loading of MB into NanoGO sheets once its characteristic absorption peak at ~ 660 nm is visible on sample NanoGO-MB. **C** Methylene blue release kinetics from NanoGO under different pH conditions on PBS 10% FBS. The higher amount of MB released in a more acidic pH results from a weaker electrostatic interaction between the negatively charged carboxylic acid groups from NanoGO and the positively charged MB. **D** On the TEM image, arrows indicate the boundaries of the graphene oxide nanosheets, magnification of ×100,000 (scale bar = 2 µm). **E** SEM image reveals the sheet-like shape and of GO, magnification of ×230 (scale bar = 100 µm). **F** Real time-infrared camera showing NanoGO temperature variation after irradiation with 808 nm NIR laser light. **G** Water and graphene oxide after irradiation with 808 nm NIR laser light for 10 min (fluency of 5.52 kJ cm^−2^). NanoGO temperature variation of ~ 40 °C. **H** DPBF absorbance decay of free MB and NanoGO-MB upon irradiation with 660 nm LED light

An overview of structural and morphological features of NanoGO and NanoGO-MB can be seen in Fig. 1. In the ATR-FTIR analysis was demonstrated that the carboxylation of the GO was quite successful. The ATR-FTIR spectrum of starting GO (Fig. 1A, black) confirms the presence of different oxygenated groups –O–H stretching (3184 cm^−1^); –C=O stretching (1728 cm^−1^); –C–OH bending (1423 cm^−1^); and –C–O stretching (1043 cm^−1^). While in the spectrum of the GO sample, the band ascribed to –C=O stretching was very weak, in the spectrum of NanoGO carboxylated (Fig. 1A, red) it was very strong, which attests to the effectiveness of the carboxylation process. The carboxylic groups added to NanoGO were in the protonated acidic form, since the –C=O stretching band peaks above 1700 cm^−1^ and the functional group at 2800 cm^−1^ corresponds to the symmetric stretching mode of –CH_2_– group in chloroacetic acid, which was used during the carboxylation reaction for add carboxylic acid groups to NanoGO [29–31].

UV–vis-NIR spectra (Fig. 1B) show a significant upward shift in the background when GO was carboxylated and cut into smaller NanoGO sheets. This rise in the optical absorption of NanoGO in both visible and NIR ranges, resulting in superior photothermal heating, which is advantageous for performing PTT [32, 33]. The spectrum of MB (Fig. 1B, navy blue) exhibited the characteristic peak of the n→π* transition at 660 nm, which was reproduced in the spectrum of the NanoGO-MB sample (Fig. 1B, light blue) and confirmed the successful loading of MB. There was also a red shift of the n→π* transition to 680 nm after MB was loaded to NanoGO, probably caused by charge transfer between MB and NanoGO.

The release kinetics of MB from NanoGO *(NanoGO* + *Pluronic F127 at 244* *µg/mL and MB at 49* *µg/mL in ultrapure water)* was conducted using a PBS solution containing 10% FBS, Fig. 1C. In the first 6 h, the amount of MB in the release solution at pH 5.0 was 12.5 and 5.5% for solution at pH 7.4. In the next time points, the amount of methylene blue released from NanoGO at pH 5.0 reached higher values when compared with pH 7.4 solution. Once the incubation time for the in vitro studies was 24 h, the release kinetics was conducted until this time point. At 24 h, 71.7% of MB was released in solution at pH 5.0, while in solution at pH 7.4 only 24.2% of the amount of MB was released. These differences in release kinetics can be a result of that in physiological pH the carboxylic acid groups from NanoGO are on their ionized form (–COO^−^), resulting in a stronger interaction with MB. However, when at pH 5.0, these carboxylate groups become protonated (–COOH) and the interaction is weakened, resulting in a faster release of MB from NanoGO. This pH-dependent release of MB obtained in conditions that mimic in vivo environment shed light on the suitability of the NanoGO-MB platform and its use on tumor treatments, once these more acidic conditions can be found in the intracellular lysosomes, endosomes and in tumors and could favor the MB release in these environments [11].

The sheet-like structure of NanoGO could be identified by TEM and SEM images provided in Fig. 1D, E, respectively [34]. The sheets transparency was confirmed by both techniques. The in vitro temperature increase of the NanoGO suspension under 808 nm NIR laser light irradiation was measured by the IR thermal imaging (Fig. 1F). Ten minutes of laser irradiation was sufficient to increase the NanoGO suspension temperature by 40 °C, reaching a maximum of 62 °C (Fig. 1G). This increase was in accordance with thermal ablation hyperthermia temperatures, which should be above 47 °C [4, 35].

Singlet oxygen production is a critical step in PDT, and its production was probed by two methods, the first one was 1,3-diphenylisobenzofuran (DPBF) absorbance and the second was electron paramagnetic resonance spectroscopy (EPR) in vitro. In DPBF method, its absorbance decreases in the presence of ROS. Under irradiation at 660 nm, the free MB solution (10 µg mL^−1^) produced a significant amount of ROS, which was lowered when MB was conjugated to NanoGO (200 µg mL^−1^ GO, 10 µg mL^−1^ MB) (Fig. 1H). After 15 min of irradiation, the ROS production by NanoGO-MB was approximately 50% of that produced by free MB, which still makes the NanoGO-MB platform suitable for use in PDT studies [36]. As expected, NanoGO alone produced no ROS. Nonetheless, graphene and GO are known to quench excited states. Once MB is excited, part of its energy is transferred to NanoGO instead of oxygen and, consequently, the ROS production is limited [11, 37]. Therefore, the lower ROS production by NanoGO-MB in vitro was expected. NanoGO-MB behavior in a biological environment, both in cells and in vivo assays, allowed a much higher ROS production since MB is being released from the NanoGO in an acidic environment. These findings confirm that the combination of NanoGO and MB is a suitable platform to be used as an agent in combined PDT/PTT therapy.

### Cell viability studies and phototoxicity of NanoGO-MB

**Fig. 2:**
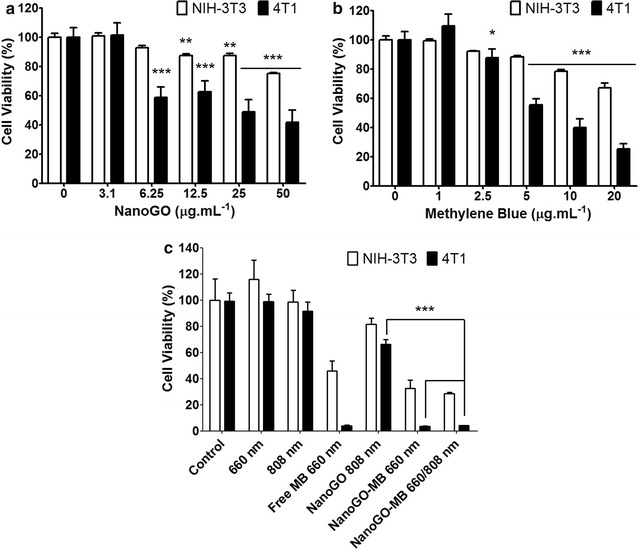
Viability of NIH/3T3 and 4T1 cells after treatment in the dark and upon irradiation. **a**, **b** NIH/3T3 and 4T1 cell viability after 24 h of exposure to NanoGO and MB in the dark, respectively. *p* values were calculated by ANOVA two-way test. **c** Combined PDT/PTT treatments in both cell lines using NanoGO-MB (NanoGO 12.5 µg mL^−1^ and MB 2.5 µg mL^−1^) as a photodynamic and photothermal agent. *p* values were calculated by the Student’s t test. Mean ± SD (n = 4) of three independent experiments, *p < 0.05, **p < 0.01, ***p < 0.001

The dark toxicity of NanoGO and free methylene blue were determined by standard MTT screening assay. Tumor and normal cells were treated for 24 h with NanoGO at concentrations of 3.1, 6.25, 12.5, 25, and 50 µg mL^−1^ and free MB at 1, 2.5, 5, 10, and 20 µg mL^−1^. NanoGO exhibits toxicity to tumor cells at all concentrations, except at 3.1 µg mL^−1^ (Fig. 2a). Free MB at concentrations of 5, 10, 20 µg mL^−1^ was toxic to both tumor and normal cells, displaying a higher toxicity in tumor cells, (*p* < 0.001) (Fig. 2b). Such toxicity can be attributed to the faster metabolism of these cells, which increases MB uptake, as in the study of Sahu et al., where they observed a higher uptake of a similar platform of NanoGO and MB by HeLa cells than by NIH/3T3 cells using confocal microscopy [11]. NanoGO-MB phototoxicity at concentrations of GO 12.5 and MB 2.5 µg mL^−1^ were verified by a cell viability assay performed using NIH/3T3 and 4T1 cells upon irradiation (Fig. 2c). Laser-only treatment did not show any impact on normal or tumor cell viability (*p* > 0.05). Meanwhile, only LED light treatment displayed an increase in tumor cell viability (*p* < 0.05). The expressive cell viability reduction of 97% in PDT and PDT/PTT groups demonstrated the potential of the proposed therapies.

Here, NanoGO-MB showed the same efficiency in promoting cell death as free MB when used with PDT only or with combined PDT/PTT. There was no statistical significance between the PDT only using free MB or PDT only and combined PDT/PTT using NanoGO-MB (*p* > 0.05). Also, PTT only treatment presented higher cell viability than 70% for both cells studied. On the other hand, in the same study conducted by Sahu et al., the authors found a statistical significance between PDT only and PTT only and combined PDT/PTT groups, while the present study did not find those differences (Fig. 2c) [11]. Based on the ROS production experiment, wherein the ROS produced by free MB was 5 times higher than platform NanoGO-MB at 5 min of irradiation (Fig. 1h), it was expected a higher viability decrease for the cells treated with free MB than to NanoGO-MB. However, comparing the cell death promoted by both treatments, upon 660 nm LED light irradiation, there was no statistical significance between them (*p* > 0.05), Fig. 2c. Therefore, the efficiency of MB loaded NanoGO in produce ROS and, subsequently, in promoting cell death was preserved showing the potential of the nanodevice since MB ability in acting as a photodynamic agent remained intact. As above mentioned, the high efficiency of NanoGO-MB platform may be the result of the protonation of the –COO^−^ groups on NanoGO under the influence of the more acidic intracellular environment (pH 5.0). Once that the electrostatic interactions between the –COOH from NanoGO and the positively-charged MB become weak, MB is released, and the quenching process mediated by graphene oxide stops, thus, giving space to ROS production by NanoGO-MB [11, 12].

### In vitro ROS production after photodynamic and/or photothermal therapy

**Fig. 3:**
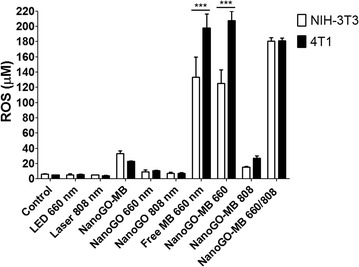
ROS production in NIH/3T3 and 4T1 cells after PDT only or PTT only and PDT/PTT combined treatments. A higher ROS production in micromolar was observed in tumor cells (*p* < 0.001) for PDT treatments only. There was no statistical significance comparing groups PDT or PTT only and PDT/PTT combined treatments for tumor cells. Data are represented as mean ± SEM (n = 3). *p* values were calculated by the Student’s t test. ***p < 0.001

For ROS detection in vitro CMH spin probe was added to cells culture before irradiation. CMH readily reacts with ROS to produce stable nitroxide radical CM^**·**^, that can be quantitatively measured by EPR. ROS production was found to be higher in tumor cells (p < 0.001) for PDT only. There was no statistical significance comparing groups PDT only or PTT only and PDT/PTT combined treatments for tumor cells. The ROS amount produced by NIH/3T3 and 4T1 cells was bellow than 40 µM for all groups, except for three groups: free MB irradiated with LED 660 nm, NanoGO-MB irradiated with LED 660 nm and NanoGO-MB irradiated with both LED 660 and 808 nm NIR light. In those groups, the ROS production reached values up to 41.4-fold times higher than the control. The higher ROS values for 4T1 cells in the groups free MB irradiated with LED 660 nm light and NanoGO-MB irradiated with LED 660 nm light can be due to a higher uptake of the nanoplatform for these cells, compared with the normal cells NIH/3T3. In addition, the similar ROS production of free MB and NanoGO-MB reveals the reestablishment of MB potential in produce ROS even when associated to NanoGO. In both cells, for combined therapies, the ROS production was not statistically significant (Fig. 3).

### Combined PDT/PTT therapies and imaging in orthotopic syngeneic model

**Fig. 4:**
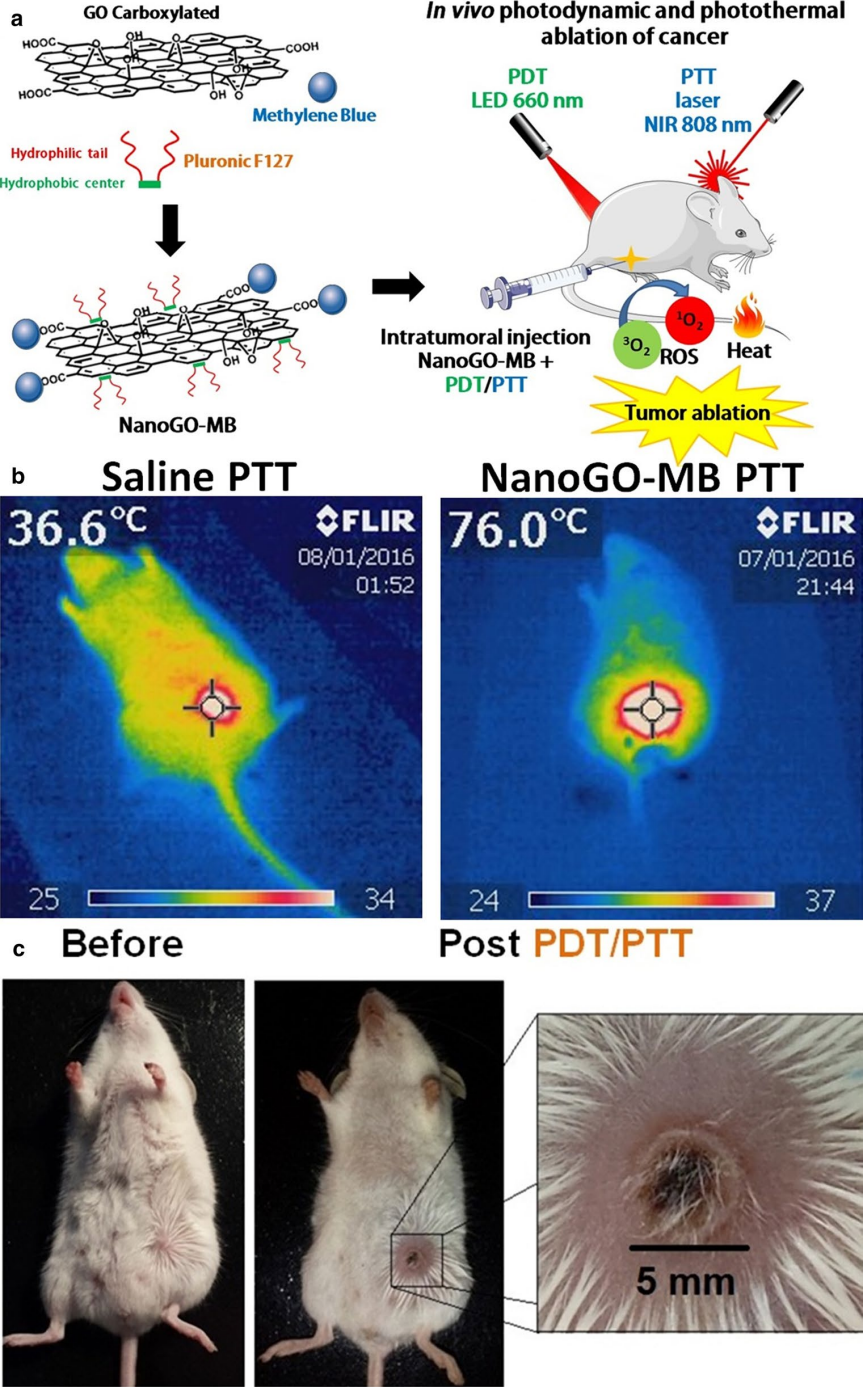
Combined PDT/PTT treatments and real-time thermal camera imaging. **a** Schematic representation of phototherapies being performed after NanoGO-MB synthesis and its intratumoral administration. On both therapies, only the tumor region was exposed to the irradiation, thus the other areas were protected from the light. **b** Real-time thermal camera imaging during PTT therapy in 4T1-Luc bearing mice showing a temperature increase of approximately 40 °C. **c** Tumor site after combined PDT/PTT therapies. The healing tissue measured 5 mm

NanoGO-MB capacity for promoting primary tumor ablation was tested upon irradiation with 660 nm LED (fluency of 90.8 J cm^−2^) and/or 808 nm NIR laser (fluency of 8.3 kJ cm^−2^) lights for 10 and 15 min, respectively (Fig. 4a). The real-time infrared camera was used to register the temperature increase on the tumor site after 3 min of irradiation of mice treated with saline or NanoGO-MB. For mice that were drug-administered and irradiated, temperatures reached up to 70 °C, as shown in Fig. 4b. Here, it was clear that the ability of NanoGO plays both the role of carrying the photosensitizer methylene blue and acts as a photothermal agent, reaching temperatures higher than 47 °C, ideal for tumor ablation [4, 35]. In mice, where there was no extravasation of NanoGO-MB during administration in solid breast tumor to the proximal tissues, such high temperatures were reached. Conversely, when NanoGO-MB appeared poorly concentrated on the tumor site, lower temperatures (between 50 and 70 °C) were reached. Due to the solid nature of the tumors, sometimes NanoGO-MB administration was not ideally performed.

The intratumoral route of administration chosen for this study was an attempt to overcome the disadvantages of tail vein i.v. injection, such as reticuloendothelial system uptake and low time blood circulation. Moreover, as suggested by Huang et al. [39], the preferred route of administration for phototherapies is intratumoral, because it concentrates the photothermal agent in a specific area, concentrating its effects [38, 39]. In groups that received PTT, on the following day after treatment, there was an appearance of a burn-related wound caused by irradiation (Fig. 4c). In some mice, PDT caused an inflammatory process at the irradiation site, and in the combined PDT/PTT groups, PTT seemed to aid in the healing process, promoting wound cauterization.

**Fig. 5:**
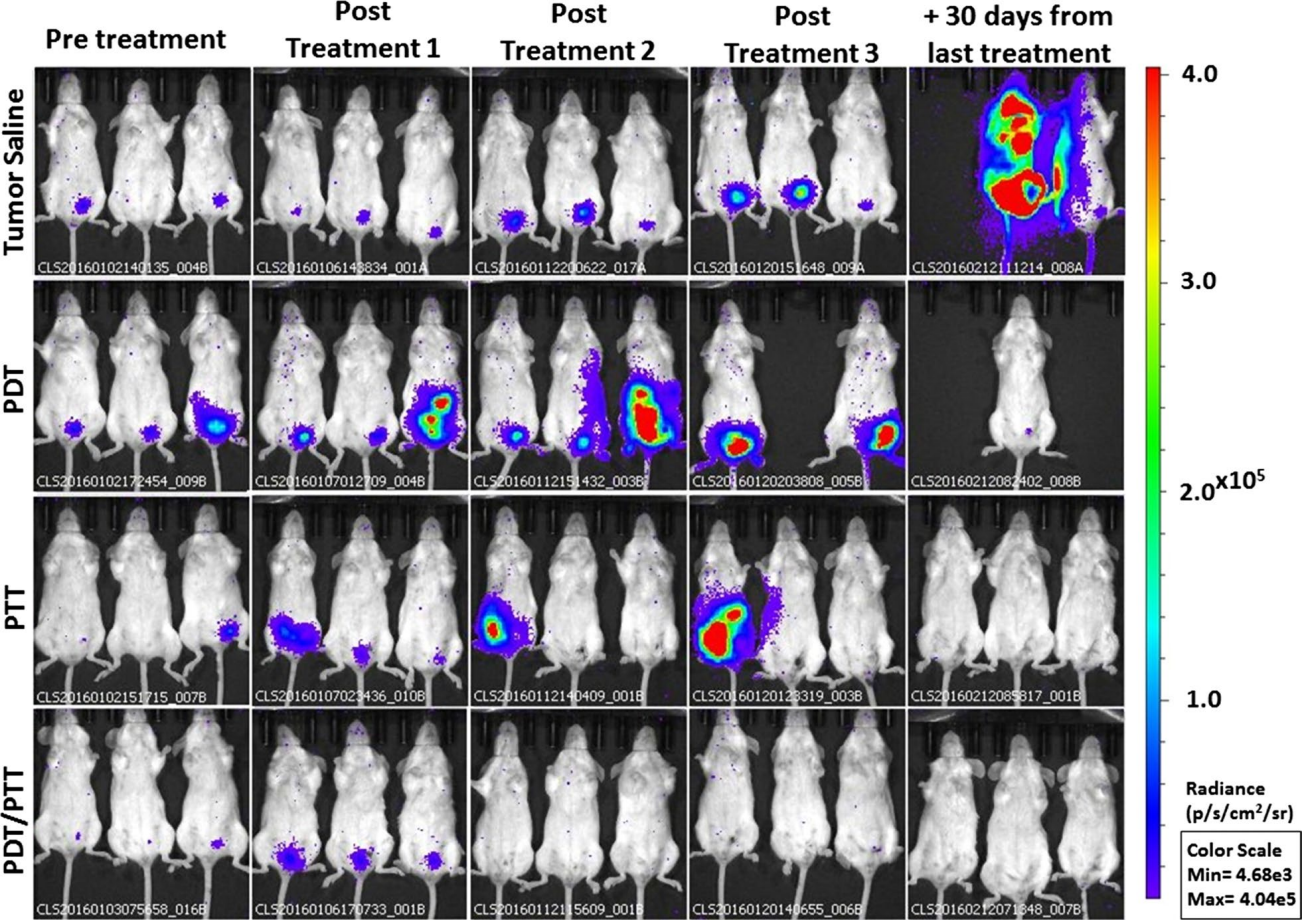
In vivo bioluminescence images. Bioluminescence signal after completion of each treatment: saline only, PDT only, PTT only and PDT/PTT combined. The bioluminescence signal reduction suggests a possible tumoral remission during the treatments on the 4T1-Luc cells-bearing BALB/c mice (n = 5). The bioluminescence signal increase on groups PDT only and PTT only over the three treatments displayed tumor growth and metastasis, whereas in the combined PDT/PTT treatment group there was a bioluminescence signal reduction throughout the treatments. Due to the color scale, the first animal from tumor saline group, in the column 30 days from the last treatment, had its bioluminescence signal overestimated. Control group (not shown) do not presented bioluminescence signal. *PDT* photodynamic therapy (660 nm LED light); *PTT* photothermal therapy (808 nm NIR laser light)

The 4T1-Luc cells in mice as a tumor model are able to mimic human breast cancer regarding tumor growth and metastasis. This kind of tumor represents an animal model for the late stage of disease, comparable to stage IV of human breast cancer [25–27, 40]. Also, in this study, the 4T1-Luc cells were used as an imaging tool since they emit bioluminescence, which is collected to monitor tumor progression and track treatment efficiency [41]. Compared with photodynamic or photothermal therapy only in vivo assays, combined PDT/PTT therapies demonstrated the synergistic effect provided by NanoGO-MB in the treatment of 4T1-Luc cell-bearing female BALB/c mice. Conversely, the in vitro results showed the same outcome for combined PDT/PTT therapies mediated by NanoGO-MB compared with only free MB, raising questions about the graphene oxide present in the nanoplatform. On the contrary, in the in vivo study, the graphene oxide nanosheets were determinant in the combined PDT/PTT therapy treatment to achieve tumor ablation without tumor regrowth after the first treatment and up to 30 days from the last treatment (Fig. 5), and metastasis prevention in major organs like liver, lungs and spleen, which did not occur in other groups what presented at least one organ affected by metastasis. In addition, low systemic toxicity was observed in pathologic examinations of liver, spleen, lungs, and kidneys.

Even in the combined PDT/PTT mice group, in which animals received two therapies, mice survived up to 30 days after the last treatment with no tumor remission, while the other groups presented metastasis in major organs and posterior death. The temperature rise caused by 808 nm NIR laser light irradiation in the presence of NanoGO-MB promoted vascular permeability changes in the vessels and tissues, increasing the blood flow at the site, and partially mitigating hypoxia [4]. This led to higher tissue oxygenation and a subsequent higher ROS production in the tissue when under irradiation by 660 nm LED light, since the ROS production relies on the amount of oxygen available in the tissue, resulting in metastasis prevention and tumor regression [4]. The 4T1-Luc cell-bearing mice were randomly grouped in the following way (n = 5 for each group): control, tumor-bearing mice treated with saline, NanoGO-MB treated with 660 nm LED light, NanoGO-MB treated with 808 nm NIR laser light and NanoGO-MB treated with both 660 and 808 nm emitting sources. The groups that received only LED, only Laser and only NanoGO-MB were omitted (see Additional file 2: Figure S1). The mice have been treated a total of three times every 4 days, and the bioluminescence was collected on each following day.

**Fig. 6:**
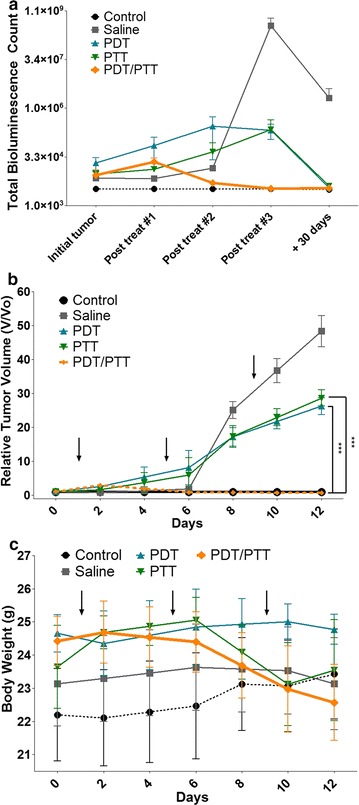
Total bioluminescence counts, relative tumor volume and body weight assessment of treated mice. **a** Tumor growth curves of different 4T1-Luc-bearing mice groups after treatments number 1, 2 and 3 (post treat #1, post treat #2 and post treat #3, respectively). The tumor volumes were normalized to their initial sizes. Statistical significance between the saline only, PDT only and PTT only groups due to the evident reduction of bioluminescence on the combined PDT/PTT group (n = 5). **b** The growth of 4T1-Luc tumors in different groups of mice during and after treatment. p value was calculated by the Student’s t test, ***p < 0.001. **c** Mean mice body weights from different groups after treatment. Arrows indicate when treatments and irradiations were performed. All data are presented as mean ± SD

For mice from the combined PDT/PTT group, after the first treatment, there was a decrease in the bioluminescence signal and, consequently, of the tumor size, whereas in the other groups the signal increased over the time. The sharp reduction of the bioluminescence signal in the saline, only PDT and only PTT groups after the third treatment was due to the deaths of one, three and one animal of those groups, respectively, by tumor progression (Fig. 6a). Most of the animals remained alive throughout the study period (approximately 60 days), even after the last imaging study. From the eight groups studied (n = 5), nine animals died due to tumor progression. Apart from the five animals mentioned above, the other four were from the following groups: tumor + 660 nm LED light group (three deaths) and tumor + 808 nm NIR light group (one death).

The relative tumor volume data was very similar to the bioluminescence total counts data, showing the same consistency in exhibiting no tumor regrowth after the first treatment in the combined PDT/PTT group, whereas, in the PDT or PTT only groups, the tumor growth persisted (p < 0.001) (Fig. 6b). There was no statistically significant variation in mice weight between the groups during the study as can be seen in Fig. 6c. However, the mice from the combined PDT/PTT group underwent more expressive weight loss than the other ones, which may be a result of the impact of the two combined therapies on them.

### Primary tumor ablation and metastasis prevention: histological and immunohistochemical analysis

**Fig. 7:**
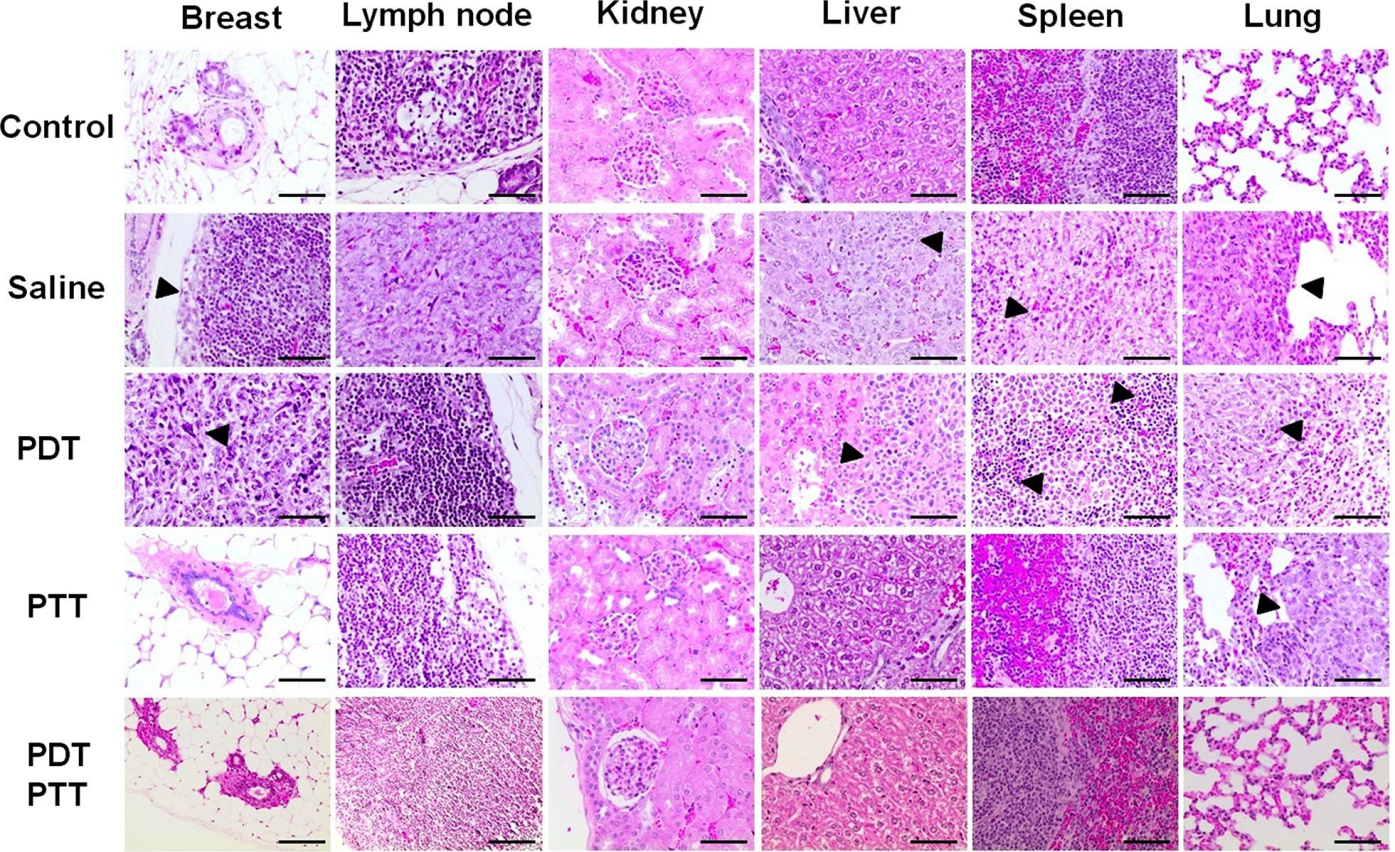
Tumor ablation and organs metastasis prevention. In the histological sections, total breast tumor ablation in PTT only and combined PDT/PTT therapies (with metastasis prevention in the major organs). The arrowheads indicate a tumor in the breast or metastasis in different organs (×400, scale bar = 100 µm)

It has been shown that the 4T1-Luc cell line has metastatic behavior similar to the parental 4T1 cell line, presenting metastasis into bone, lungs, liver, and brain, organs primarily affected in human breast cancer [25]. Concordantly, the histological results showed metastasis in liver, lungs, and spleen (Fig. 7). In the breast tumor site, a complete loss of tissue pattern was observed, with angiolymphatic invasion in the PDT only group. However, none of the groups presented satellite lymph node metastasis, with the metastasis possibly moving through the blood vessels instead of lymphatic vessels. Death by metastatic breast cancer is still a major concern even with all clinical advances [1, 2].

**Fig. 8:**
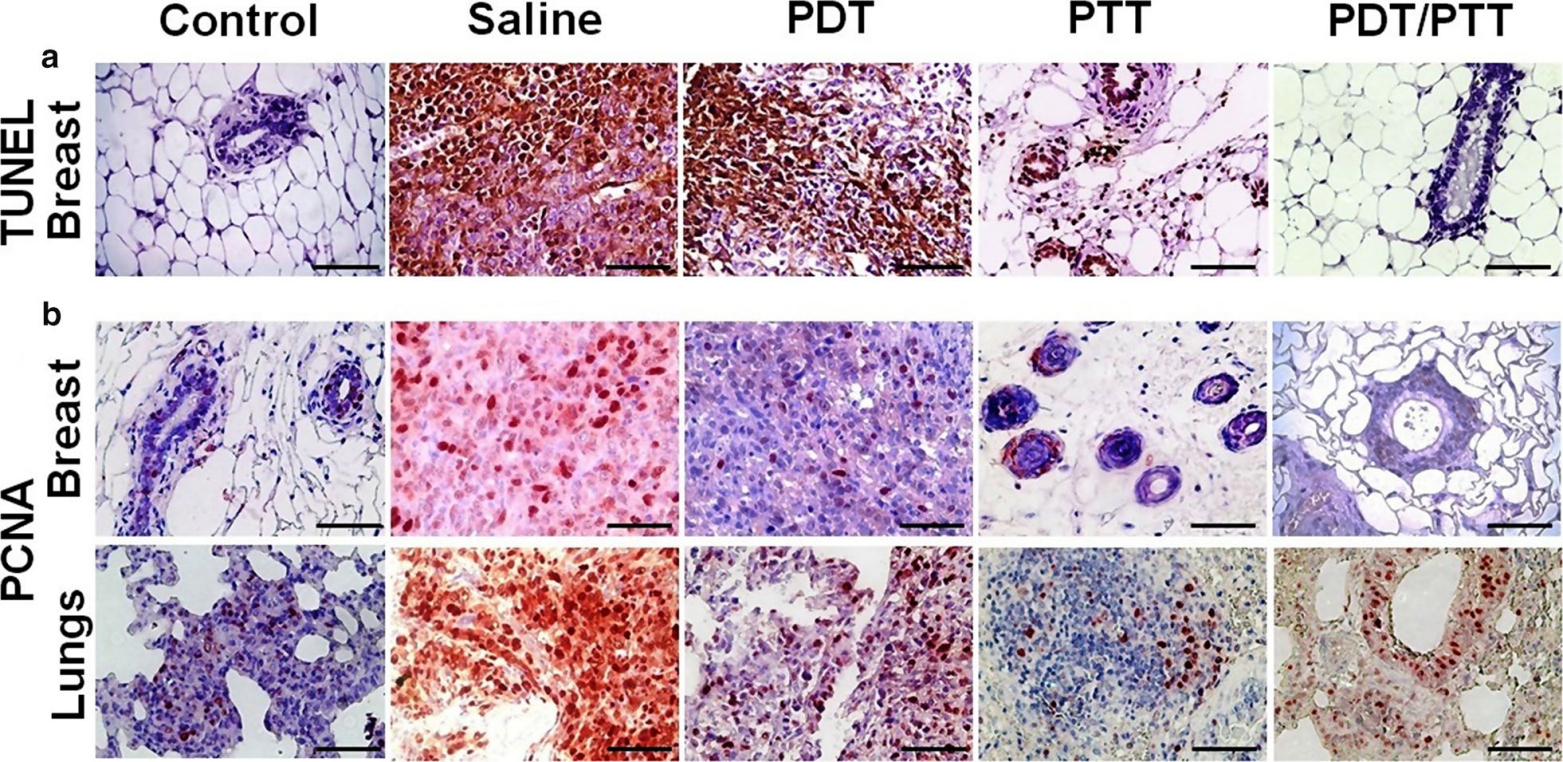
In situ apoptosis detection by TUNEL staining and immunolocalization of PCNA positive cells. The brown dye colored cells represent both TUNEL (control and tumoral breast tissues) and PCNA positive cells (breast and lung tissues). The sections were prepared 30 days from the last mice treatment with the phototherapies. **a** For TUNEL, only in breast tissue, these positive cells can be observed mainly in the saline group without an expressive labeling of cells for the combined PDT/PTT group. **b** Proliferating cell nuclear antigen labeled cells were natively found in lung tissue even in the control group. Breast tissue presented higher labeling in the saline group and lower in the treated groups, PDT and PTT only and combined PDT/PTT therapies, suggesting the potential of the therapies. Magnification ×400, scale bar = 100 µm. There was no statistical significance between the groups (*p* > 0.05)

In this study, combined PDT/PTT treatment promoted primary tumor ablation and stopped its progression. In addition, metastasis appeared to be hindered after combined phototherapy administration. Conversely, the saline and PDT only groups presented severe metastasis in liver, spleen, and lungs. Nevertheless, the treatment of the PTT group was efficient in promoting tumor ablation (Fig. 7). In a deeper analysis, PDT only, PTT only and combined PDT/PTT treatment groups presented necrotic cells at the tumor site, which may be a sign of therapy efficacy [42]. However, increased necrosis was found at PDT only and PTT only group when compared with combined PDT/PTT treatment groups, showing that the combined therapies provided a cleaner treatment, since necrosis is undesirable. To verify the lack of occurrence of metastasis in the combined PDT/PTT group, as seen in the bioluminescence imaging and histological analysis, the slides were treated for immunodetection of apoptotic (TUNEL) and proliferating (PCNA) cells. In Fig. 8, the brown dye colored cells labeled for TUNEL revealed lower levels of apoptosis for groups treated with PTT only and combined PDT/PTT treatment. Although PCNA analyses in lungs revealed a high cellular proliferation rate in the saline, PDT and PTT only groups. Our analyses revealed indexes of PCNA-positive cells did not differ between treatments groups and negative control. PCNA, TUNEL and histological findings suggest that combined PDT/PTT induces a lesser morphological injury.

According to the statistical test performed, there was no statistical significance (*p* > 0.05) between the groups or in the different techniques or organs studied. Nevertheless, it was clear that the amount of brown dye colored cells in the combined PDT/PTT group was lower than the saline, PDT, and PTT only groups, resulting in a visual aspect similar to the control group for the breast with TUNEL or PCNA techniques.

For future studies, the delivery of a NanoGO-MB platform could be improved by a polyethylene glycol coating, which would allow graphene oxide to associate with antibodies or peptides in order to enable a tumor-targeted delivery increasing its specificity in vivo.

## Conclusion

The developed NanoGO-MB platform has shown the capacity to promote complete tumor ablation without regrowth and with metastasis prevention upon the combined PDT/PTT in metastatic breast tumor in a murine model. Owing to the strong photoabsorption of nanographene oxide sheets and ROS production by MB, all tumors in the combined PDT/PTT therapy group were functionally defeated upon exposure to LED and NIR laser light sources. Thus, this suggests the potential of NanoGO-MB for the treatment of breast cancer in preclinical studies.

## Materials and methods

### Materials

Graphite flakes (0.45 mm), sodium nitrate, sodium hydroxide, monochloroacetic acid, potassium permanganate, sulfuric acid (98%), hydrochloric acid (36–37%), hydrogen peroxide (30%), and methylene blue were all purchased from Sigma-Aldrich, USA, and used as received. Pluronic F127 (Mw 12,600 g mol^−1^) from BASF Corp. (USA) was used without additional purification. Murine fibroblasts (NIH/3T3) and murine mammary carcinoma (4T1 cells) were purchased from American Type Culture Collection (ATCC, USA) and Cell Bank of the Rio de Janeiro (Rio de Janeiro, Brazil), respectively. 4T1-Luc cells were obtained by cotransfecting 4T1 cells while using Lipofectamine PLUS with firefly luciferase-containing pGL-3-control vector and the puromycin resistance vector, pKO-puro, according to the Invitrogen (USA) protocol at Nanobiotechnology Laboratory of the University of Brasilia. Diethyldithiocarbamic acid sodium salt (DETC), deferoxamine methanesulfonate salt (DF), 1-hydroxy-3-methoxycarbonyl-2,2,5,5-tetramethylpyrrolidine (CMH), 3-carboxy-2,2,5,5-tetramethyl-1-pyrrolidinyloxy (CP^·^), and Krebs HEPES buffer (KHB) were purchased from Noxygen (Elzach, Germany).

Dulbecco’s modified Eagle’s medium (DMEM) and Roswell Park Memorial Institute (RPMI) 1640 cell culture medium, heat-inactivated fetal bovine serum (FBS), and penicillin/streptomycin, trypsin and 3-(4,5-dimethylthiazol-2-yl)-2,5-diphenyltetrazolium bromide (MTT) were purchased from Gibco (NY, USA). Phosphate-buffered saline and dimethyl sulfoxide were purchased from Laborclin (Paraná, Brazil) and Sigma-Vetec (São Paulo, Brazil), respectively. All water used was the ultrapure type (18 MΩ cm), provided by a Barnstead™Easypure™ II purification system from Thermo Scientific (CA, USA). Dialysis of NanoGO suspensions was performed with an MWCO 12 kDa cut-off dialysis bags from Sigma-Aldrich (MO, USA). A second purification step was carried out by centrifugation (5000 rpm) with Amicon Ultra-15 Centrifugal Filter Units cut-off MWCO 100 kDa, purchased from Millipore (MA, USA). PDT and PTT experiments were conducted with a setup designed and built at the Laboratory of Software and Instrumentation in Applied Physics at the University of Brasilia-UnB.

The system is provided with a 660 nm LED light and an 808 nm NIR laser light. The PDT equipment was composed of a 660 nm LED model GP-100Wr6-G42M-Z3GL, from Green Powertech Solutions Limited. The power was controlled using pulse width modulation (PWM) applied to a 480 Hz square wave. The temperature was controlled via a commercial water cooler Corsair H95 actuating as a heat sink attached to the high-power LED. The 808 nm NIR laser was driven by a DC power supply Agilent model E4356A. In both equipments, the irradiance calibration was performed using a FIELD MAX II Energy and Power meter, item # 1098580, serial # 0099L11R from Coherent, obtaining individual data from each point in the range of application.

### Methods

#### Nano graphene oxide (NanoGO) preparation

NanoGO was prepared in a four-step synthetic route, which comprised: (i) preparation of graphene oxide by the Hummers and Offeman procedure [43–45]; (ii) ultrasonic exfoliation of graphitic oxide in ultrapure water to produce GO; (iii) carboxylation of GO with mono chloroacetic acid and sodium hydroxide; and (iv) ultrasonic stirring. Steps (i) and (ii) were performed according to the procedure detailed elsewhere [46]. The carboxylation step (step iii) was carried out according to the procedure described by Sahu et al. [11]. In brief, 10 mL of GO suspension (1 mg mL^−1^, sodium hydroxide (200 mg; 0.005 mol) and monochloroacetic acid (150 mg; 0.0016 mol) were all mixed in a round bottom flask (125 mL), transferred to a water bath and heated at 45 °C for 4 h under magnetic stirring. The resulting suspension of carboxylated GO, or GO-COOH, was then transferred to a dialysis bag (12,000) and dialyzed against ultrapure water, for 2 days, with periodic changing of water.

After dialysis, the GO-COOH suspension was stirred with an ultrasonic probe Q700 Sonicator (QSonica, USA) at 500 W, in the pulse mode to prevent heat buildup, for 4 h in order to reduce the size of the sheets and produce NanoGO. The resulting NanoGO suspension was submitted to successive spin centrifuge filtration at 5000 rpm using an Amicon Ultra-15 MWCO 100 kDa centrifugal filter and washed with ultrapure water. This procedure was repeated several times until the suspension reached neutral pH. The neutral NanoGO suspension was submitted to an additional ultrasonic stirring step for 2 h at 500 W and then filtered through a 0.22-µm pore membrane filter to remove large aggregates. The filtered suspension was stored in a fridge at 10 °C for further characterization and biological tests.

#### Pluronic stabilization of NanoGO and methylene blue loading

The colloidal stability of NanoGO was improved further by mixing, with the aid of magnetic stirring, 10 mL of NanoGO suspension (0.2 mg mL^−1^) with 20 mg of Pluronic F127. After mixing, the homogeneous suspension was incubated at 4 °C for complete dissolution of the polymer. Shortly after, the suspension was ultrasonicated for 30 min at 500 W and incubated at 37 °C to promote adsorption of Pluronic F127 onto the NanoGO surface. Unbound free Pluronic F127 was removed by spin centrifuge filtration using an MWCO 100 kDa filter. The MB loading onto Pluronic F127-modified NanoGO was performed by addition of an MB stock solution. The mixture was then kept at 37 °C for 1 h under magnetic stirring. After 1 h, unbound free MB was removed by spin centrifuge filtration through an MWCO 100 kDa filter. The final PDT/PTT platform, called hereafter NanoGO-MB, refers to carboxylated nanographene oxide sheets associated with Pluronic F127 and loaded with MB. The term Pluronic F127 was omitted from the name for clarification.

#### Structural and morphological characterization of NanoGO and NanoGO-MB

The electronic structures of NanoGO and NanoGO-MB, as well as confirmation of MB loading, were assessed by UV–vis absorption spectroscopy (Lambda 35 UV–vis spectrometer, PerkinElmer, USA). Absorption spectra were registered in the range of 450–900 nm with a 10-nm slit width. The effectiveness of the carboxylation reaction was evaluated by Fourier transform infrared (FTIR) spectroscopy. FTIR spectra were acquired with a continuum Fourier transform infrared microscope attached to the FTIR 6700 bench (Thermo Fisher, USA) in attenuated total reflection (ATR) mode (32 scans and resolution of 2 cm^−1^). The hydrodynamic diameter and zeta potential of NanoGO samples were assessed by dynamic light scattering (DLS) and electrophoretic mobility, respectively, with a Zetasizer Nano ZS instrument (Malvern Instruments, UK). All measurements were carried out at 25 °C using ultrapure water as the solvent. NanoGO ultrastructure was analyzed by transmission electron microscopy (TEM) (JEOL JEM 1011, JEOL, Japan) at 100 kV and scanning electron microscopy (SEM) (JEOL JSM-700 1F, JEOL, Japan) at 15 kV.

#### Analysis of methylene blue release kinetics from NanoGO

NanoGO-MB platform (500 µL) was placed in MWCO 12 kDa cut-off dialysis bags and immersed in 25 mL of phosphate-buffered saline containing 10% of fetal bovine serum (FBS) in pH 5.0 or pH 7.4. The bags and solutions of different pH were kept at 37 °C in a shaking platform (100 rpm). The analysis of the MB release rate from NanoGO was carried out collecting samples at 1, 6, 12, 18 and 24 h time points. At every solution change, whole release medium was replaced by the new fresh medium. The amount of MB released from NanoGO was assessed by measuring its absorbance at the peak of maximum absorption of 660 nm by an UV–vis spectrophotometer. A standard calibration curve with a known concentration of MB in PBS 10% FBS was used to calculate the exact concentration of the release solutions. The results were expressed in percentage of released MB comparing with the initial amount of MB added to the NanoGO suspension.

#### Singlet oxygen generation

The singlet oxygen (^1^O_2_) generation by free MB and NanoGO-MB was measured by monitoring the absorbance bleaching of the 1,3-diphenylisobenzofuran (DPBF) [22, 47]. For the assay, individual sample aliquots (200 µL) of free MB (10 µg mL^−1^), NanoGO (as a control, 200 µg mL^−1^) and NanoGO-MB (200 µg mL^−1^ GO, 10 µg mL^−1^ MB) were placed in a 96-well plate and 10 µL of ethanolic DPBF solution (225 µg mL^−1^) was added to each sample. The samples’ absorbance at 410 nm (DPBF max) were registered in a spectrophotometer (SpectraMax M2e, Molecular Devices, CA, USA), before and after irradiating for 30 s with 660 nm LED light (power density ~ 150 mW cm^−2^). The results were expressed as the DPBF absorbance (%).

#### Photothermal activity of NanoGO

Aliquots (200 µL) of diluted NanoGO suspension (200 µg mL^−1^) were placed in a 96-well plate and then irradiated with an 808 nm NIR laser light, with a spot size of 0.26 cm^2^ and power density of 9.2 W cm^−2^. The plate was irradiated for 10 min and the light-induced temperature change on water and in NanoGO suspension was monitored as a function of the irradiation elapsed time every minute by means of a type K thermocouple placed on the suspension. The thermocouple was kept away from the point of laser incidence to minimize direct heating. The temperature change was also monitored in real-time with an infrared thermal imaging system (FLIR SC-300, FLIR Systems Inc, Danderyd, Sweden).

#### Dark and phototoxicity assays

Experiments were carried out using a normal murine fibroblast cell line (NIH/3T3) and murine mammary carcinoma cells (4T1 cells). The NIH/3T3 and 4T1 cells were cultured in DMEM and RPMI 1640 cell culture medium, respectively, containing 10% of heat-inactivated FBS and 1% antibiotic (penicillin–streptomycin) at 37 °C in an 80% humid CO_2_ incubator. The cell viability under dark conditions was determined using different concentrations of the NanoGO suspension (3.1, 6.25, 12.5, 25 and 50 µg mL^−1^) and free MB solution (1, 2.5, 5, 10 and 20 µg mL^−1^) for 24 h, without LED or NIR laser light treatments. Cells (3 × 10^4^ per well) were seeded into 96-well cell culture plates and grown for 24 h at 37 °C. Afterward, cells were exposed to treatments for 24 h and were protected from light exposure. After incubation, fresh medium containing 0.5 mg mL^−1^ of MTT (3-(4,5-dimethylthiazol-2-yl)-2,5-diphenyltetrazolium bromide) was added. Cells were further incubated for 2 h at 37 °C. Formazan crystals were solubilized with 200 mL of dimethyl sulfoxide, and the medium absorbance was measured at 595 nm using a spectrophotometer (Spectra-Max M2e, Molecular Devices, CA, USA). The same measurement procedure was repeated for samples submitted to photodynamic and/or photothermal therapy performed by irradiation for 3 min with LED and NIR laser light, respectively. Cells were seeded as previously, and 24 h later, a cell medium solution containing free MB (2.5 µg mL^−1^), NanoGO (12.5 µg mL^−1^) and NanoGO-MB (12.5 µg mL^−1^ of NanoGO and 2.5 µg mL^−1^ of MB) replaced the seeding media. The plates were incubated for 24 h to allow cell uptake. Then, without a washing step, in an attempt to simulate the in vivo conditions, PDT groups were treated with 660 nm LED light for 3 min (fluency of 34 J cm^−2^), whereas PTT groups were irradiated with 808 nm NIR laser light also for 3 min (fluency of 1.65 kJ cm^−2^). In the combined PDT/PTT group, cells were first submitted to 660 nm LED light followed by 808 nm NIR laser light irradiation at the same treatment conditions described above. Cells were incubated for an additional 24 h and viabilities were measured by MTT assay as previously described.

#### In vitro ROS production after photodynamic and/or photothermal therapy

Cells NIH/3T3 and 4T1 (3 × 10^4^ per well) were seeded into 24-well cell culture plates and grown for 24 h at 37 °C. Afterward, cells were exposed to the treatments as on the phototoxicity assay for 24 h, protected from light exposure. The ROS sensitive spin probe CMH (stock solution 10 mM prepared in KHB containing 25 µM DF and 5 µM DETC to minimize the oxidation of CMH by Fenton reaction due to transition metals) was added to a final concentration of 250 µM in 600 µL cell culture medium before the irradiation with LED 660 nm and/or 808 nm NIR laser lights. Then, the cells were irradiated for PDT or PTT only and PDT/PTT combined therapies and allowed to stand at 37 °C for 1 h. After that, 450 µL of supernatant was transferred to a 1 mL de-capped syringe and snap frozen in liquid nitrogen. All the samples were stored at − 80 °C until the EPR measurements were performed. EPR measurements were performed in a Bruker spectrometer (Bruker EMXplus, Germany), equipped with an X-band (9 GHz) high sensitivity cavity (Bruker ER 4119HS, Germany). For ROS detection, the samples were transferred to a liquid nitrogen dewar (Noxygen, Germany) and the spectra were recorded at 77 K. The instrumental settings were 2 mW microwave power, 5 G amplitude modulation, 100 kHz modulation frequency and 200 G sweep width. The peak to peak amplitude, meaning the distance between the lowest and the highest points in the first derivative spectrum, was used for detection of the signal. A calibration curve was obtained using the nitroxide radical (CP^·^) diluted in KHB to the following concentrations: 0, 10, 50, 100, 250 e 500 µM. In this concentration range, a linear calibration curve was obtained and all the recorded data were within this calibration range.

#### In vivo photodynamic and photothermal therapy in a syngeneic orthotopic tumor model

Female BALB/c mice (8 weeks old, 21–25 g) were acquired from the Institute of Energy and Nuclear Research (São Paulo, Brazil). Mice under anesthesia were orthotopically implanted with 4T1-Luc cells (2 × 10^4^ cells in 50 µL of DMEM medium without serum) by subcutaneous injection at a 90° angle to the nipple of the fifth left breast of each animal. Treatments started when tumors reached ~ 25 mm^3^.

Mice were randomly divided into the following eight experimental groups (five mice per group):

1. Control without tumor;
2. Tumor + saline;
3. Tumor + NanoGO-MB;
4. LED: tumor + 660 nm LED light;
5. Laser: tumor + 808 nm NIR laser light;
6. PDT: tumor + NanoGO-MB + 660 nm LED light;
7. PTT: tumor + NanoGO-MB + 808 nm NIR laser light; and
8. Combined PDT/PTT: tumor + NanoGO-MB + 660 nm LED light + 808 nm NIR laser light.

Only phosphate buffer saline (PBS) was administered to Groups 1 and 2. All other groups were intratumorally injected with NanoGO-MB (25 µL, dose 10 mg kg^−1^ of NanoGO and 2.5 mg kg^−1^ of MB) through the mouse nipple, as detailed above. Ten minutes after the NanoGO-MB was administered, anesthetized mice received irradiation for PDT (660 nm LED light for 10 min, fluency of 90.8 J cm^−2^), PTT (808 nm NIR laser light for 15 min, fluency of 8.3 kJ cm^−2^) and combined PDT/PTT therapies for 10 and 15 min, respectively, at the tumor site. During irradiation, the non-interest regions were protected from LED and NIR laser light irradiation. For group 8, the treatment for PTT was performed after the PDT therapy. In total, each mouse received three treatments every 4 days. During the NIR laser irradiation, the real-time temperature change at the tumor site was monitored by an infrared thermal imaging system. Pre and post-treatment tumor sizes were measured at specific time points by a digital caliper and calculated as volume = (tumor length) × (tumor width)^2^/2. The obtained value expressed as relative tumor volumes were calculated as V/V_0_ (V_0_ was the tumor volume when the treatment was initiated).

#### Progression of tumor growth, metastasis and imaging in vivo

The 4T1-Luc tumor-bearing mice were monitored every 4 days with bioluminescence images (BLI). For this, 100 µL of d-luciferin (10 mg mL^−1^, at a dose of 10 mg kg^−1^, PerkinElmer, USA) in PBS were intraperitoneally injected and, after 10 min, animals were imaged under anesthesia with 1.5% isofluorane in an IVIS Lumina XR In Vivo Imaging System (Caliper LifeSciences, USA). A total of 20 BLI (60 s of exposition each) were collected before and after each treatment using a field of view of 12.5 cm. The emission was collected at 560 nm with a time-correlated single photon counting system. The bioluminescence calculation was made taking into account the background removal, i.e. final bioluminescence was an equal region of interest (ROI) emitting bioluminescence signal minus the background of a different area with the same ROI size (n = 5).

#### Histology

Shortly after the last bioluminescence analysis (30 days from the last treatment), mice were euthanized. A full necropsy was performed, and tumors of the left breast and satellite lymph nodes, liver, lung, spleen and kidney were excised. The tissues were fixed with 4% paraformaldehyde, dehydrated in ethanol, sectioned into 5.0 µm sections and processed for histology. Hematoxylin & Eosin staining of paraffin-embedded tissues was used for histological examination of primary tumors, organs and eventual metastasis. The sections were analyzed and photographed using an Olympus Vanox-T microscope at 400× magnification and an Olympus U-PMTVC with CCD camera.

#### In situ apoptosis detection by TUNEL staining

Apoptotic cell death in breast deparaffinized tissue sections was detected using terminal deoxynucleotidyl transferase-mediated digoxigenin deoxyuridine nick-end labeling (TUNEL) with an ApopTagR Plus Peroxidase In Situ Apoptosis Kit (Chemicon, USA). Sections were treated with proteinase K (20 mg mL^−1^, Dako) in PBS (0.01 M, pH 7.4) for 15 min at room temperature. Endogenous peroxidase was inactivated by 3% H_2_O_2_ (10 min). Another blockage was performed with 6% skim milk in PBS (0.01 M, pH 7.4) for 30 min at 37 °C. Slides were incubated with the enzyme working strength TdT in a wet chamber for 1 h at 37 °C and then incubated with anti-digoxigenin for 30 min at room temperature.

Finally, sections were treated with 3,30-diaminobenzidine for 30 s and counterstained with Mayer’s hematoxylin. As a negative control, a kidney section was incubated with 1.0 U mL^−1^ DNase, whereas for positive control a section of spleen tissue was used. Positive nuclei of apoptotic cells were identified by a dark brown color under a light microscope at 400× of magnification in ten different fields. The degree of apoptotic cells was calculated as a percentage of labeled cells.

#### Immunohistochemical detection of proliferating cell nuclear antigen—PCNA

PCNA expression in deparaffinized and rehydrated tissue sections was analyzed by immunostaining. Antigen exposure on sections was obtained by treatment for 20 min using target retrieval system (DAKO, CA, EUA). The activity of endogenous peroxidase was blocked by immersing sections in 3% hydrogen peroxide (H_2_O_2_) in 50% methanol. For reduction of nonspecific signals in the reaction, sections were then treated with blocking buffer (6% skim milk in PBS, pH 7.4) for 30 min at 37 °C. Incubation of sections with the primary antibody IgG2 anti-PCNA (Dako, CA, EUA), at a dilution of 1:1300, was carried out overnight at 4 °C. For conjugation with the secondary antibody, sections were treated with EnVision-HRP (horseradish peroxidase complex) (DAKO, CA, EUA) following the manufacturer’s specifications.

Sections were incubated with 3-amino-9-etil-carbazol (AEC) (DAKO, CA, EUA) for antibody conjugation with peroxidase, and then counterstained with Carazzi’s hematoxylin, also for antibodies with peroxidase. All stained tumor sections were imaged with light microscopy under 400× magnification. The analysis consisted of quantification of positive cells per field. The degree of PCNA expression was calculated as the percentage of antibody-labeled cells.

#### Statistical analysis

Analysis of variance (ANOVA) (two-way) was performed, followed by Bonferroni post hoc test for multiple comparisons and Student’s t test using the software GraphPad Prism 5.0 (CA, EUA). Statistical significance was defined as *p < 0.05, **p < 0.01 and ***p < 0.001. Data were presented as the mean ± standard deviation. The statistical analysis of the immunohistochemical data was performed using the software R. Normality of data was verified with the Shapiro–Wilk test, followed by data analysis using Kruskal–Wallis test.

## Additional files

**Additional file 1: Table S1.** Nanomaterials Colloidal Stability and Properties. The colloidal stability of different platforms, NanoGO, NanoGO + Pluronic, NanoGO-MB, and NanoGO-MB + Pluronic, prepared in different media (deionized water, phosphate buffer saline, pH 7.4, and Dulbecco’s Modified Eagle’s Medium (DMEM) with 10% of FBS was ascertained by measuring their hydrodynamic diameter with dynamic light scattering. The measurements were taken after right after of samples preparation.

**Additional file 2: Figure S2.** Bioluminescence images of LED or NIR laser light irradiation only and NanoGO-MB only treated groups. The increase in the bioluminescence signal indicates tumoral progression and the absence of some 4T1-Luc-bearing mice during the treatments means the death of these individuals during the experiment.
