# Supplementary material for: Nanographene oxide-methylene blue as phototherapies platform for breast tumor ablation and metastasis prevention in a syngeneic orthotopic murine model
Source: J Nanobiotechnology. 2018 Jan 30;16:9. doi: 10.1186/s12951-018-0333-6 (PMC5789561; doi:10.1186/s12951-018-0333-6)
Supplement: Supplementary file 1 — Additional file 1: Table S1. Nanomaterials Colloidal Stability and Properties. The colloidal stability of different platforms, NanoGO, NanoGO + Pluronic, NanoGO-MB, and NanoGO-MB + Pluronic, prepared in different media (deionized water, phosphate buffer saline, pH 7.4, and Dulbecco’s Modified Eagle’s Medium (DMEM) with 10% of FBS was ascertained by measuring their hydrodynamic diameter with dynamic light scattering. The measurements were taken after right after of samples preparation. [file 12951_2018_333_MOESM1_ESM.doc]

RESEARCH

# Nanographene Oxide-Methylene Blue as Phototherapies Platform for Breast Tumor Ablation and Metastasis Prevention in a Syngeneic Orthotopic Murine Model

Mayara Simonelly Costa dos Santos, Ana Luisa Gouvêa, Ludmilla David de Moura, Leonardo Giordano Paterno, Paulo Eduardo Narcizo de Souza, Ana Paula Bastos, Emanuel Adelino Medeiros Damasceno, Fabiane Hiratsuka Veiga-Souza, Ricardo Bentes de Azevedo and Sônia Nair Báo**^*^**.

| **Sample/ Diluent** | **DH (nm)** | **PDI** |
| --- | --- | --- |
| NanoGO/water | 200.08 ± 2.61 | 0.328 ± 0.02 |
| NanoGO/PBS | 1353.33 ± 156.84 | 0.495 ± 0.15 |
| NanoGO/DMEM | 363.60 ± 10.90 | 1.00 ± 0.00 |
| NanoGO/RPMI | 123.00 ± 3.90 | 0.64 ± 0.14 |
| NanoGO Pluronic F127/water | 188.93 ±3.59 | 0.300 ± 0.03 |
| NanoGO Pluronic F127/PBS | 1096.33 ± 60.47 | 0.269 ± 0.05 |
| NanoGO Pluronic F127/DMEM | 112.71 ± 16.59 | 0.820 ± 0.16 |
| NanoGO Pluronic F127/RPMI | 104.4 ± 1.35 | 0.70 ± 0.01 |
| NanoGO MB/water | 10473.00 ± 7879.06 | 0.571 ± 0.42 |
| NanoGO MB/PBS | 868.50 ± 314.62 | 0.183 ± 0.11 |
| NanoGO MB/DMEM | 79.10 ± 54.21 | 0.370 ± 0.07 |
| NanoGO MB/RPMI | 30.50 ± 8.43 | 0.645 ± 0.14 |
| NanoGO Pluronic F127 MB/water | 4733.33 ± 1491.64 | 0.826 ± 0.15 |
| NanoGO Pluronic F127 MB/PBS | 5436.00 ± 1655.20 | 0.732 ± 0.23 |
| NanoGO Pluronic F127 MB/DMEM | 137.57 ± 41.19 | 0.401 ± 0.05 |
| NanoGO Pluronic F127 MB/RPMI | 224.27 ± 34.19 | 0.716 ± 0.17 |

##

## The colloidal stability of different platforms, NanoGO, NanoGO+Pluronic, NanoGO-MB, and NanoGO-MB+Pluronic, prepared in different media (deionized water, phosphate buffer saline, pH=7,4, and Dulbecco's Modified Eagle's Medium (DMEM) with 10% of FBS was ascertained by measuring their hydrodynamic diameter with dynamic light scattering. The measurements were taken after right after of samples preparation.
