# Supplementary material for: Nanographene oxide-methylene blue as phototherapies platform for breast tumor ablation and metastasis prevention in a syngeneic orthotopic murine model
Source: J Nanobiotechnology. 2018 Jan 30;16:9. doi: 10.1186/s12951-018-0333-6 (PMC5789561; doi:10.1186/s12951-018-0333-6)
Supplement: Supplementary file 2 — Additional file 2: Figure S2. Bioluminescence images of LED or NIR laser light irradiation only and NanoGO-MB only treated groups. The increase in the bioluminescence signal indicates tumoral progression and the absence of some 4T1-Luc-bearing mice during the treatments means the death of these individuals during the experiment. [file 12951_2018_333_MOESM2_ESM.doc]

RESEARCH

# Nanographene Oxide-Methylene Blue as Phototherapies Platform for Breast Tumor Ablation and Metastasis Prevention in a Syngeneic Orthotopic Murine Model

Mayara Simonelly Costa dos Santos, Ana Luisa Gouvêa, Ludmilla David de Moura, Leonardo Giordano Paterno, Paulo Eduardo Narcizo de Souza, Ana Paula Bastos, Emanuel Adelino Medeiros Damasceno, Fabiane Hiratsuka Veiga-Souza, Ricardo Bentes de Azevedo and Sônia Nair Báo**^*^**.

##
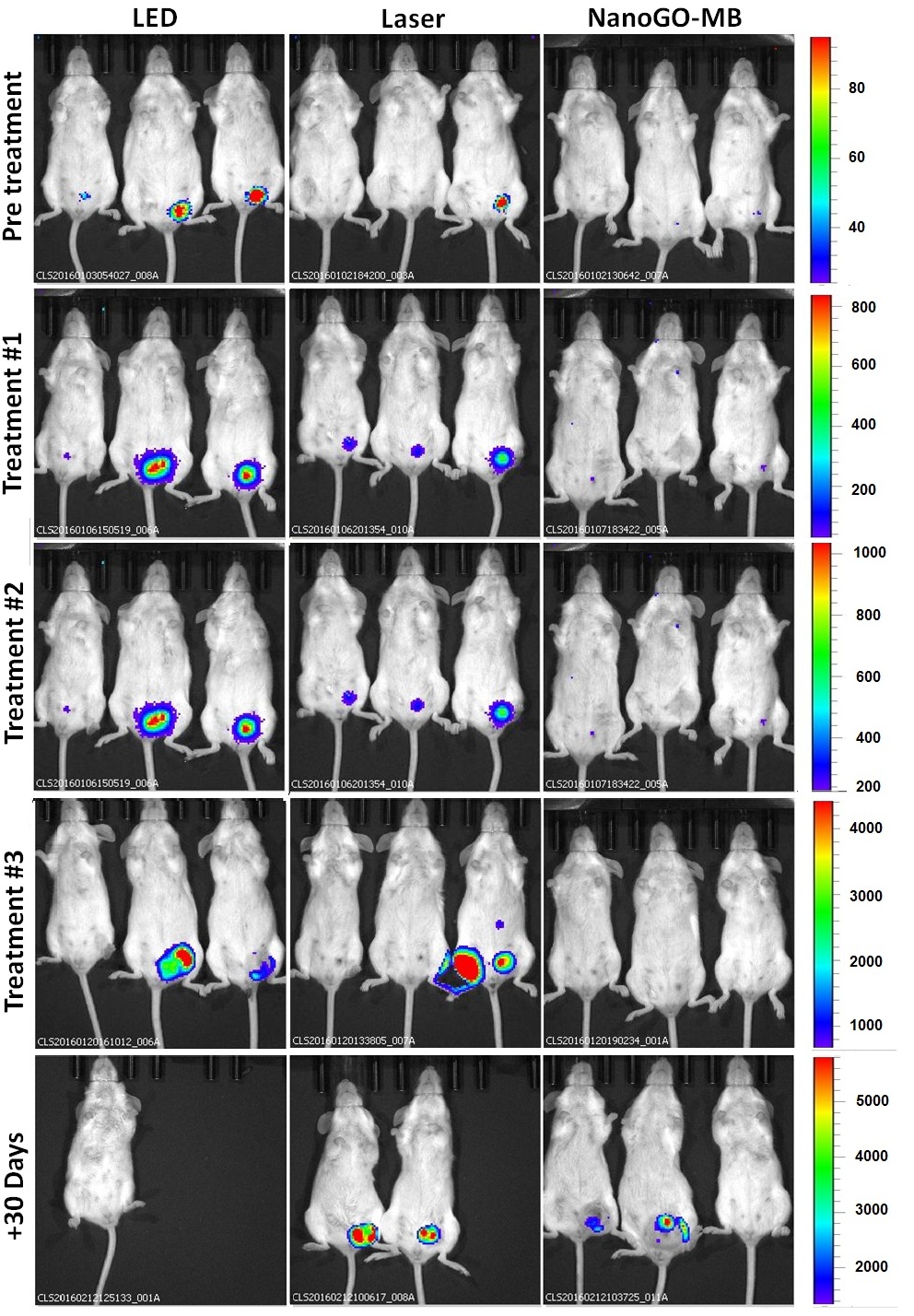
Bioluminescence images of LED or NIR laser light irradiation only and NanoGO-MB only treated groups. The increase in the bioluminescence signal indicates tumoral progression and the absence of some 4T1-Luc-bearing mice during the treatments means the death of these individuals during the experiment.
